# Humoral Hypercalcemia of Malignancy Caused by Parathyroid Hormone-Related Protein-Secreting Medullary Thyroid Carcinoma: A Case Report

**DOI:** 10.70352/scrj.cr.25-0546

**Published:** 2026-03-18

**Authors:** Takuya Hirose, Hirotaka Nakayama, Osamu Matsubara, Satoru Uchida, Kazuyuki Tani, Nobuyasu Suganuma, Aya Saito

**Affiliations:** 1 Department of Surgery, Hiratsuka Kyosai Hospital, Hiratsuka, Kanagawa, Japan; 2 Department of Surgery, Yokohama City University, Yokohama, Kanagawa, Japan; 3 Department of Pathology, Hiratsuka Kyosai Hospital, Hiratsuka, Kanagawa, Japan; 4 Department of Endocrinology and metabolism, Hiratsuka Kyosai Hospital, Hiratsuka, Kanagawa, Japan

**Keywords:** medullary thyroid carcinoma, hypercalcemia, malignancy, parathyroid hormone-related protein

## Abstract

**INTRODUCTION:**

Medullary thyroid carcinoma (MTC) is a rare neuroendocrine tumor arising from the parafollicular C cells, accounting for approximately 1.5% of all thyroid cancers. Although calcitonin secreted by the MTC typically lowers calcium levels, serum calcium concentrations usually remain within the normal range due to compensatory parathyroid hormone (PTH) secretion. Hypercalcemia of malignancy is broadly categorized as humoral hypercalcemia of malignancy (HHM), mediated by parathyroid hormone-related protein (PTHrP), or local osteolytic hypercalcemia. We report a rare case of HHM caused by a PTHrP-secreting MTC.

**CASE PRESENTATION:**

A 60-year-old woman visited our hospital with weight loss, fatigue, and a large right-sided neck mass. Laboratory tests revealed marked hypercalcemia (16.5 mg/dL), hypophosphatemia, and renal dysfunction. The intact PTH level was within the normal range, whereas the PTHrP level was elevated to 5.7 pmol/L, consistent with HHM. Tumor marker analysis revealed marked increases in carcinoembryonic antigen and calcitonin levels. Imaging studies revealed a large mass in the right thyroid lobe without evidence of regional or distant metastasis. Fine-needle aspiration confirmed the diagnosis of MTC. Genetic testing was negative for rearranged during transfection mutations and multiple endocrine neoplasia 2A, supporting a sporadic form. Hypercalcemia was managed with intravenous hydration (saline solution, 2 L/day for 10 days), elcatonin (80 units/day for 9 days), and a single dose of zoledronic acid. The patient underwent a right thyroid lobectomy. Histopathological analysis confirmed MTC without extra-thyroidal extension or lymph node metastasis (Union for International Cancer Control pT3aN0M0 stageII). Postoperatively, the serum PTHrP levels decreased to normal, and the patient recovered without complications. At the 8-month follow-up, no evidence of recurrence was observed.

**CONCLUSIONS:**

Herein, we present a rare case of MTC that caused hypercalcemia via PTHrP production. Although HHM is uncommon in thyroid cancer, the condition can cause severe hypercalcemia requiring prompt diagnosis and treatment. HHM should be considered in patients with thyroid cancer with hypercalcemia, and PTHrP measurement may aid in the diagnosis.

## Abbreviations

ATC
: anaplastic thyroid carcinoma
CA
: carbohydrate antigen
CEA
: carcinoembryonic antigen
FDG-PET/CT
: 18F-fluorodeoxyglucose PET/CT
FECa
: fractional excretion of calcium
FHH
: familial hypocalciuric hypercalcemia
HHM
: humoral hypercalcemia of malignancy
IL
: interleukin
LOH
: local osteolytic hypercalcemia
MEN2A
: multiple endocrine neoplasia 2A
MTC
: medullary thyroid carcinoma
PHPT
: primary hyperparathyroidism
PTC
: papillary thyroid carcinoma
PTH
: parathyroid hormone
PTHrP
: parathyroid hormone-related protein
RET
: rearranged during transfection

## INTRODUCTION

MTC is a rare malignancy originating from parafollicular cells, accounting for approximately 1.5% of all thyroid cancers.^1)^ Although serum calcitonin levels are elevated in MTC and exert a hypocalcemic effect, serum calcium levels typically remain within the normal range due to the compensatory PTH secretion.^2,3)^

However, in rare cases, tumor-derived factors can cause hypercalcemia, broadly categorized as HHM and LOH. HHM results from the systemic effects of PTHrP secreted by tumor cells, while LOH is caused by bone resorption mediated by cytokines produced locally by tumor cells within bone lesions.^4,5)^

Here, we report a rare case of HHM caused by sporadic MTC associated with elevated PTHrP production.

## CASE PRESENTATION

A 60-year-old woman was referred to our hospital for evaluation of progressive weight loss, fatigue, and a right cervical mass. The mass had been noted 10 years prior but remained uninvestigated. Her medical history included the presence of ovarian cysts and retinitis pigmentosa. She was not taking any medication and had no known allergies or relevant family history. She was a lifelong nonsmoker and abstained from alcohol consumption.

Upon initial examination, her height and weight were 164.1 cm and 49.9 kg, respectively. Her blood pressure was 200/100 mmHg, and her heart rate was 103 beats/min with a regular rhythm. Physical examination revealed a soft, mobile, and elastic mass, approximately 10 cm in diameter, in the right thyroid lobe.

**Fig. 1:**
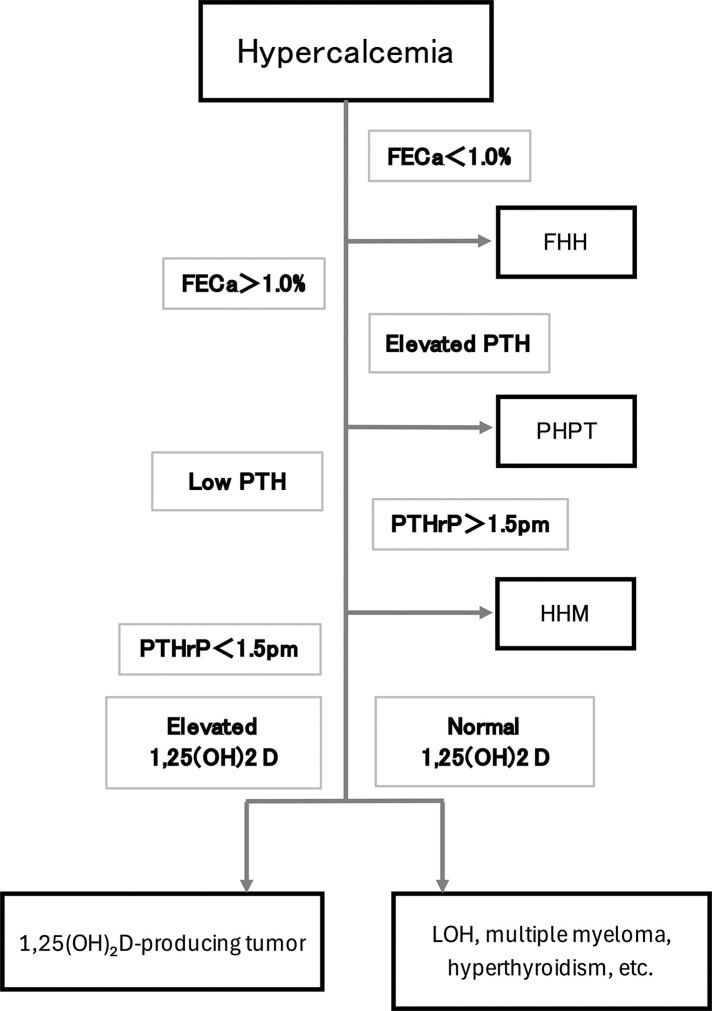
Diagnostic algorithm of hypercalcemia. FECa, fractional excretion of calcium; FHH, familial hypocalciuric hypercalcemia; HHM, humoral hypercalcemia of malignancy; LOH, local osteolytic hypercalcemia; PTH, parathyroid hormone; PTHrP, parathyroid hormone-related protein

Laboratory data revealed marked hypercalcemia (16.5 mg/dL; normal range 8.8–10.4 mg/dL), hypophosphatemia (2.3 mg/dL; normal, 2.5–4.5 mg/dL), and renal dysfunction (creatinine 2.19 mg/dL; normal, 0.47–0.79 mg/dL). Further evaluation following a diagnostic algorithm (**Fig. 1**) demonstrated elevated fractional excretion of calcium (FECa 10.7%; normal, 2%–4%), normal intact PTH (12 pg/mL; normal, 10–65 pg/mL), and elevated PTHrP (5.7 pmol/L; normal, ≤1.3 pmol/L), findings suggestive of HHM (**Table 1**). Tumor markers revealed elevated CEA (12243 ng/mL; normal, <5.0 ng/mL) and calcitonin (160 pg/mL; normal, ≤6.4 pg/mL), while thyroglobulin, CA19-9, CA125, CA15-3, and neuron-specific enolase levels were normal.

**Table 1:** Laboratory data

Hematology		Hormone	
WBC	10200/μL	TSH	0.232 μIU/mL
Neut	77.5%	fT3	2.58 pg/mL
Lymp	15.5%	fT4	1.33 ng/dL
Mono	6.5%	I-PTH	12 pg/mL
RBC	439 10^4^/μL	PTHrP	5.7 pmol/L
Hb	12.8 g/dL	ACTH	19.1 pg/mL
Plt	33.4 10^4^/μL	CORT	17.1 μg/dL
		Metanephrine	62 pg/mL
Biochemistry		Normetanephrine	132 pg/mL
ALB	4.7 g/dL		
AST	25 U/L	Tumor marker	
ALT	24 U/L	CEA	12243 ng/mL
ALP	106 U/L	Calcitonin	160 pg/mL
BUN	25 mg/dL	CA19-9	19.4 U/mL
Cre	2.19 mg/dL	CA125	14.7 U/mL
Na	142 mEq/L	CA15-3	7.7 U/mL
K	4 mEq/L	NSE	4.2 ng/mL
Cl	106 mEq/L		
Ca	16.5 mg/dL	Serology	
IP	2.3 mg/dL	CRP	0.59 mg/dL
1,25(OH)2 D	24.4 pg/mL	sIL-2R	375 U/mL
FECa	10.7%		

CEA, carcinoembryonic antigen; FECa, fractional excretion of calcium; PTHrP, parathyroid hormone-related protein

**Fig. 2:**
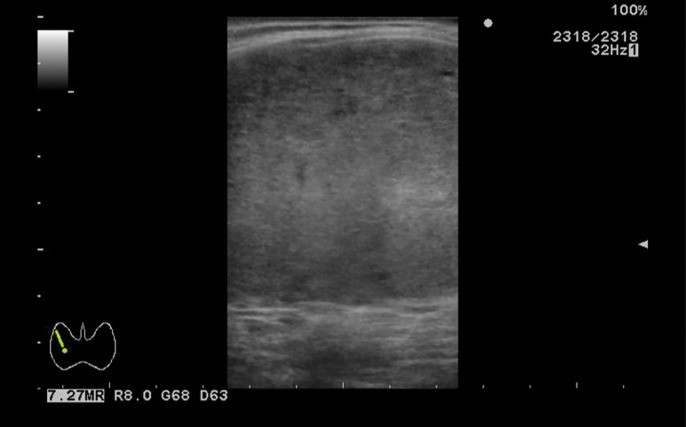
Ultrasonography. Ultrasonography revealed a hypoechoic mass measuring 100 mm in the right thyroid lobe.

**Fig. 3:**
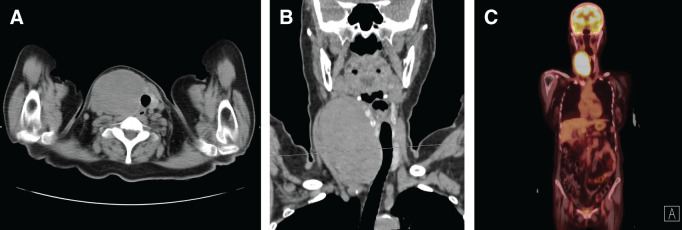
FDG-PET/CT. (**A**) CT revealed a 93-mm mass within the right thyroid lobe. (**B**) The mass did not invade the trachea. FDG-PET/CT revealed increased uptake in the mass. (**C**) No evidence of lymph node involvement or distant metastasis was observed. FDG-PET/CT, 18F-fluorodeoxyglucose PET/CT

Ultrasonography revealed a well-circumscribed, homogeneously hypoechoic mass measuring 100 mm in the right thyroid lobe (**Fig. 2**). FDG-PET/CT demonstrated increased uptake in a 93-mm mass within the right thyroid lobe, with no evidence of lymph node involvement or distant metastasis (**Fig. 3**). Upper and lower gastrointestinal endoscopies revealed no malignancy.

**Fig. 4:**
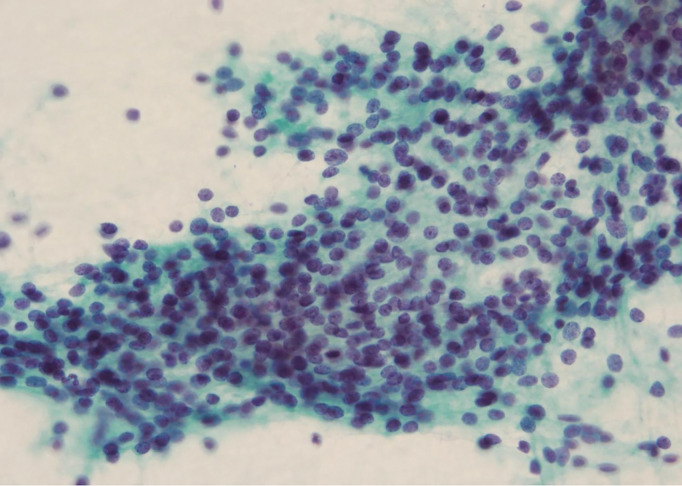
Fine-needle aspiration cytology. Fine-needle aspiration cytology of the thyroid mass revealed small round cells with scant stroma.

Fine-needle aspiration cytology of the thyroid mass revealed small round cells with scant stroma and no distinctive architectural pattern consistent with MTC (**Fig. 4**).

Plasma metanephrine and normetanephrine levels assessed for MEN2A were within normal limits (62 and 132 pg/mL, respectively). Genetic testing for RET mutations yielded negative results, confirming the diagnosis of sporadic MTC associated with HHM.

The patient initially underwent correction of the hypercalcemia with intravenous hydration (2 L/day for 10 days), elcatonin (80 units/day for 9 days), and a single dose of zoledronic acid (4 mg). After biochemical improvement, a right thyroid lobectomy with unilateral central neck lymph node dissection was performed.

The operative time was 163 min, and the intraoperative blood loss was minimal (3 mL). No gross invasion into adjacent structures or lymph node metastasis was observed. The final surgical stage was sT3aN0.

**Fig. 5:**
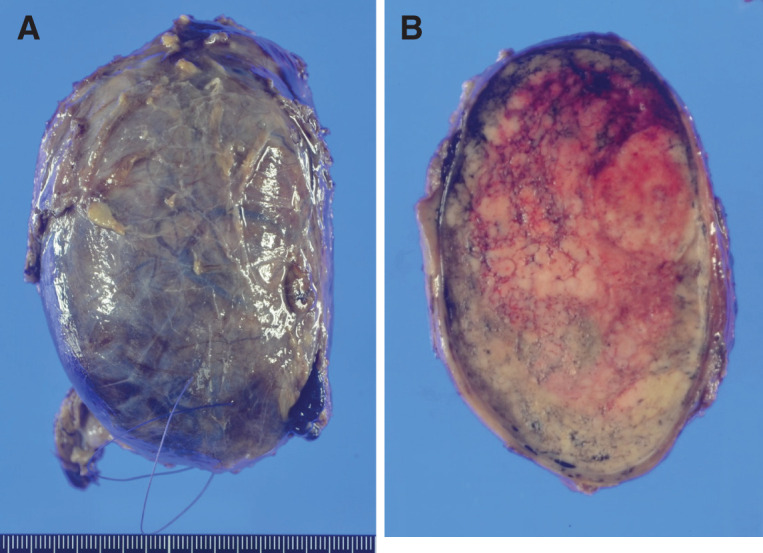
The resected specimen. (**A**) The resected specimen was a solitary nodule of the right lobe of the thyroid, 11 x 7 x 7 cm in size, with a smooth surface. (**B**) On section was a brown to yellow tumor mass appearance with hemorrhages.

**Fig. 6:**
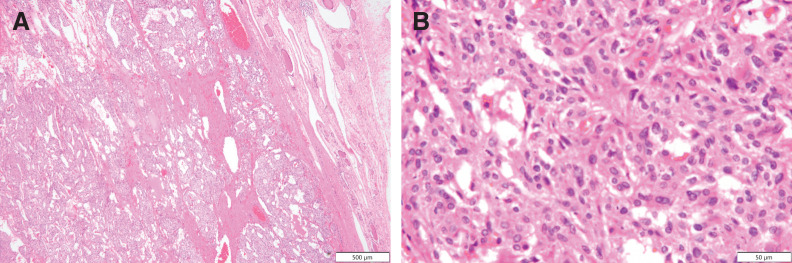
Hematoxylin and Eosin staining. The tumor tissue consists of (**A**) sheets and solid nests of (**B**) round and polygonal cells with pale eosinophilic cytoplasm.

**Fig. 7:**
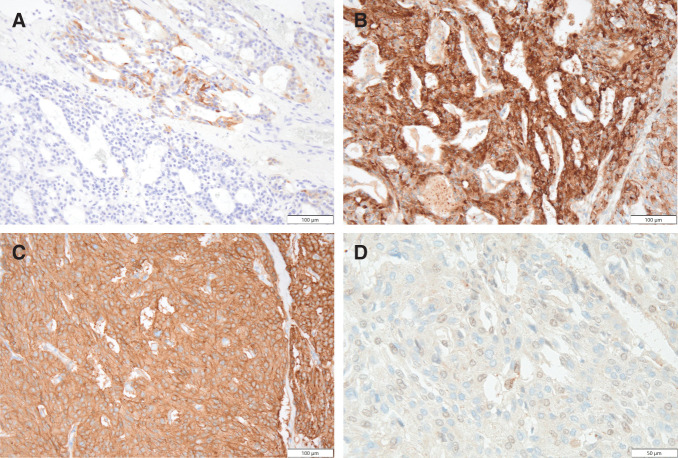
The tumor cells were (**A**) partially positive for calcitonin, and (**B**) positive for chromogranin A, (**C**) synaptophysin, CD56, CEA, and TTF1. The tumor cells were (**D**) positive for PTHrP in the nuclei and cytoplasm. CEA, carcinoembryonic antigen; PTHrP, parathyroid hormone-related protein

The resected specimen was a solitary nodule of the right lobe of the thyroid, 11 x 7 x 7 cm in size, with a smooth surface (**Fig. 5A**). On section was a brown to yellow tumor mass appearance with hemorrhages (**Fig. 5B**). Microscopically, the tumor tissue consists of sheets and solid nests of round and polygonal cells with pale eosinophilic cytoplasm (**Fig. 6A** and **6B**). The tumor did not contain prominent nucleoli, multinucleated tumor cells, or nuclear grooves or inclusions. Amyloid deposition or calcification was not seen. Immunohistochemically the tumor cells were partially positive for calcitonin (**Fig. 7A**) and positive for chromogranin A (**Fig. 7B**), synaptophysin, CD56, CEA (**Fig. 7C**), and TTF1. Based on these findings, a diagnosis of MTC was confirmed. In addition, the tumor cells were positive for PTHrP in the nuclei and cytoplasm (**Fig. 7D**).

Immunohistochemical staining for PTHrP was performed using a mouse monoclonal PTHLH/PTHrP antibody (catalog no. MAB6734; Bio-Techne/R&D Systems, Minneapolis, MN, US) at a dilution of 1:25.

Additionally, extrathyroidal extension or lymph node involvement was absent. The pathological stage was T3aN0M0 stage II according to the Union for International Cancer Control (UICC) classification, 8th edition.

Serum PTHrP levels decreased to <1.1 pmol/L POD 2. The patient recovered uneventfully and was discharged on POD 4. Postoperatively, CEA and calcitonin levels exhibited a sustained decline, eventually normalizing. Eight months post-surgery, the patient remained disease-free with no clinical or biochemical recurrence.

## DISCUSSION

Several conditions can cause hypercalcemia, including PHPT, HHM, LOH, and FHH. Among malignancy-related cases, HHM and LOH represent the two major subtypes, with HHM accounting for approximately 80%.^4)^

In HHM, PTHrP secreted by tumor cells exerts systemic effects, promoting osteoclastic bone resorption and increasing renal tubular calcium reabsorption, ultimately leading to hypercalcemia. Endogenous PTH levels are typically suppressed by negative feedback in this setting. The diagnostic hallmarks of HHM include hypercalcemia, hypophosphatemia, elevated PTHrP levels, and suppressed PTH levels.

Conversely, LOH is caused by the local production of osteoclast-activating cytokines such as IL-1, IL-6, and tumor necrosis factor within metastatic bone lesions, leading to increased osteoclastic bone resorption and subsequent hypercalcemia.^6)^

In the present case, following the diagnostic algorithm illustrated in **Fig. 1**, low-normal intact PTH, elevated FECa, and increased PTHrP levels confirmed the diagnosis of HHM.

Representative tumors known to cause HHM include adult T-cell leukemic lymphoma, squamous cell carcinomas (of the lungs, head and neck, and skin), renal cell carcinoma, and breast cancer.^7)^ However, HHM associated with thyroid carcinoma is rare.

Kitamura et al. reported that among 127 patients who died of thyroid cancer, 8 (6.3%) developed hypercalcemia, with 6 of these (75%) attributed to HHM.^5)^ Among the HHM cases, five were ATC and one was PTC.

A literature search for “thyroid cancer” and “PTHrP” in PubMed, Ichushi (Japanese medical database), and Google Scholar identified eight previously reported cases of thyroid carcinoma with measured PTHrP levels. These eight cases, along with the present case, are summarized in **Table 2**.^5,8–13)^

**Table 2:** Past cases of patients with thyroid carcinoma and HHM

	Age	Sex	PTHrP	Intact-PTHrP	Ca(mg/dL)	Histology
Kitamura^5)^	48	M	11.1 pmol/L		15.2	ATC
Yazawa^8)^	67	F	4.02 pmol/L		13.8	ATC
Kunisue^9)^	63	F	39 pmol/L		14	ATC
Iwai^10)^	84	F		3.2 pmol/L	10.9	ATC
Ito^11)^	60	F		138 pg/mL	11.2	PTC
Ito^11)^	58	F		284 pg/mL	11.4	PTC
Okutur^12)^	50	M	8.5 pmol/L		13.8	MTC
Ackah^13)^	8	F	301 pmol/L		18.8	MTC
Our Case	60	F	5.7 pmol/L		16.5	MTC

ATC, anaplastic thyroid carcinoma; F, female; HHM, humoral hypercalcemia of malignancy; M, male; MTC, medullary thyroid carcinoma; PTC, papillary thyroid carcinoma

A correlation between PTHrP and serum calcium levels has been reported in oral squamous cell carcinoma.^14)^ However, HHM associated with thyroid cancer is rare, and no definitive correlation has been established to date. Given the rarity of HHM associated with thyroid cancer, further case studies are warranted.

## CONCLUSIONS

Here, we report a rare case of MTC presenting with hypercalcemia due to PTHrP production. Although HHM is uncommon in thyroid cancer, it can cause severe hypercalcemia, highlighting the need for prompt diagnosis and treatment. In cases of thyroid cancer-associated hypercalcemia, HHM should be considered, and PTHrP measurements may aid diagnosis. Due to scarce reports, further accumulation of clinical data is required to improve our understanding of this rare condition.
